# The Challenges in the Development of Diabetes Prevention and Care Models in Low-Income Settings

**DOI:** 10.3389/fendo.2020.00518

**Published:** 2020-08-13

**Authors:** Feneli Karachaliou, George Simatos, Aristofania Simatou

**Affiliations:** ^1^Unit of Endocrinology, Diabetes and Metabolism, 3rd University Pediatric Department, Attikon University Hospital, Athens, Greece; ^2^Department of Breast Surgery, Agios Savvas Hospital, Athens, Greece

**Keywords:** diabetes mellitus, health care models, diabetes prevention, diabetes management, low and middle income

## Abstract

In low- and middle-income countries (LMICs), the burden of non-communicable diseases such as diabetes is rapidly rising, overpassing the existing burden of communicable diseases. Patients with diabetes living in low-income communities face unique challenges related to lack of awareness, difficulty in accessing health care systems and medications, and consequently failure in achieving optimal diabetes management and preventing complications. Effective diabetes prevention and care models could help reduce the rising burden by standardizing guidelines for prevention and management, improving access to care, engaging community and peers, improving the training of professionals and patients and using the newest technology in the management of the disease. In this article, we review the latest research and evidence on effective models of diabetes prevention and diabetes care delivery in low- income settings. We also provide existing evidence relating to the effectiveness of these models in low-resource contexts, with the aim to highlight characteristics and strengths that make their implementation successful and long-lasting.

## Introduction

Diabetes is one of the most prevalent non-communicable diseases associated with increased morbidity, mortality, and economic burden (1). In 2015, an estimated 415 million people were living with diabetes mellitus (DM) and the number is expected to rise to 624 million by 2040 (2). This increase is in parallel with the worldwide growing epidemic of obesity, as well as the population growth and aging (3). Diabetes is also a costly disease with great economic burden on affected people and health care systems, particularly in low- to middle-income countries (LMICs) because of its longevity and serious associated macrovascular and microvascular complications.

In 2013, 77% of patients with diabetes (382 million) lived in LMICs (2). Moreover, the projected increase in prevalence is higher in low-income countries (108%) and LMICs (60%) compared to upper-middle countries (51%) and high-income countries (HICs) (28%). The rate of increase is inversely related to the countries' income status (3) and has been attributed to the rapid urbanization followed by the adoption of sedentary lifestyles and unhealthy dietary habits (4).

At present, 7 of the top 10 countries with the largest number of people with diabetes are LMICs, including China with a prevalence of 9.2% [95% confidence interval (CI) = 8.6–11.9%); India, 10.4% (95% CI = 8.4–13.0%); Brazil, 10.4% (95% CI = 9.2–11.5%); Pakistan, 19.9% (95% CI = 8.3–30.9%); Indonesia, 6.3% (95% CI = 5.4–6.8%); and Bangladesh, 9.2% (95% CI = 7.6–11.8%) (5–9). India has the second largest number of people with type 2 DM (T2DM) (> 69 million) after China, and the largest number of individuals (36.5 million) with impaired glucose tolerance (IGT) and prediabetes (2). Most of these individuals are at high risk of developing diabetic complications (4).

High-income countries have specialists in endocrinology, related subspecialties' health care, the means to cover the costs of medications and glucose monitoring devices, and the funds to support diabetes research and public health initiatives. Many LMICs experience lack of endocrinology specialists and clinical staff shortages, poor laboratory facilities, and limitations in diabetes treatments and supplies. Lack of public awareness about diabetes and its comorbidities affects patients' engagement with health care services and adherence to prescribed treatment. Economic difficulties challenge the affordability of medications and medical supplies by many patients (10).

To minimize these obstacles, many organizations, institutions, and researchers in western countries have tried to organize global health programs and develop comprehensive models of diabetes care specifically designed for a particular LMIC and/or low-resource health care settings, or introduce new concepts in the existing care systems (11–23). Additionally, national programs with collaborative partnerships between medical schools, hospitals, health centers, and initiatives implemented by local researchers have been attempted (24–28). Randomized controlled trials (RCTs) have been organized to determine whether some low-cost interventions in diabetes therapy might improve treatment outcomes (29, 30).

In this article, by conducting a literature review, we present diabetes care models in LMICs for preventing/delaying the onset of the disease in high-risk individuals and for the control of the disease when already present.

We used a keyword search for articles published in PubMed, using the following terms: *diabetes mellitus, LMICs, health care models, prevention models, diabetes management*. Publications in languages other than English were excluded. Only peer-reviewed studies reporting development and implementation of diabetes care prevention or management models in LMICs by World Bank grouping were characterized as relevant and included in this review. Studies in upper-middle-income and upper-income countries or studies evaluating the effect of specific medications in low income subpopulations in HICs or LMICs were excluded. This review focused on T2DM, which accounts for 90 to 95% of diagnosed cases of the disease and did not include data on gestational or specifically type one diabetes.

We also tried to identify the features and qualities that can make these programs successfully implemented, scaled up, and sustained (31) in the diabetes care in these low-resource contexts.

## Prevention Models

Several RCTs (32–36) have demonstrated the effectiveness of early diagnosis and prevention of T2DM among individuals with IGT. Intervention programs aiming at lifestyle modifications including diet, physical activity, and health behaviors have been shown to decrease the incidence of T2DM in high-risk populations in HICs even after the end of the program (36). Additionally, good glycemic control and control of multiple risk factors such as blood pressure, lipids, and so on, have been shown to reduce the incidence and severity of major diabetic complications (37).

However, interpreting evidence from HICs to LMICs is not appropriate, taking into account the significant economic and cultural differences within targeted populations.

The data on prevention trials in high-risk populations for T2DM in LMICs are limited (38–47) compared to HICs. There is successful evidence especially from China and India where large RCTs of lifestyle intervention among subjects with IGT resulted in substantial reductions in diabetes incidence among participants (38, 39). Even from 1986, when China was ranked among LMICs, the implementation of an active prevention program consisting of diet, exercise, or combination of them on 557 individuals with IGT followed over 6 years resulted in decrease in risk of developing diabetes by 31, 46, and 42%, respectively, compared to controls (38). Similarly in the Indian Diabetes Prevention Program (IDPP), lifestyle modification, metformin alone, or combined, implemented on 395 subjects with IGF followed for nearly 3 years significantly reduced the relative risk by 28.5, 26.4, and 28.2%, respectively (39).

In IDPP-3, intervention consisted of individually tailored lifestyle modification by mobile phone messaging compared to standard lifestyle advice at 10 work sites in India. A significant reduction in diabetes incidence (hazard ratio = 0.64, 95% CI = 0.45–0.92) was reported in the intervention group, implying that using mobile phones may be a useful methodology for delivering advice and education, overcoming the barriers of cost and access to at-risk subjects (40). More recently, in the same country, intervention group received lifestyle modification in information technology consisting of mobile phone and e-mail (virtual assistance)–based lifestyle advice. After 1 year, the prevalence of overweight/obesity reduced by 6.0% in the intervention group and increased by 6.8% in controls who received no educational program (risk difference 11.2%, *p* = 0.042), reinforcing the hypothesis that virtual assistance-based lifestyle intervention can be cost-effective in reducing risk factors for diabetes (41). In the Kerala Diabetes Prevention Program, an RCT conducted in 60 polling areas (clusters) of Kerala state, India, high-diabetes-risk individuals participated in a 12-month community-based peer-support program comprising 11 peer-led group sessions, 2 diabetes prevention education sessions, and participation in community activities to support lifestyle change. Participants were supplied with handbooks, workbooks, and health educational booklets. This low-cost community-based peer support lifestyle intervention resulted in significant improvements in some cardiovascular risk factors but not a significant reduction in diabetes incidence (42) Similarly in Brazil, a nutrition education program in high-risk subjects for T2DM using frequent individual and group nutritional counseling was shown to improve anthropometric, dietary and metabolic parameters in these high-risk individuals (43). In Vietnam, 417 individuals consisting of the intervention group received a nutrition and physical activity program for 6 months, which led to significant improvements in several metabolic and anthropometric parameters (44).

The Diabetes Community Lifestyle Improvement Program (45) was an RCT of diabetes prevention in adults with any form of prediabetes (IGT, impaired fasting glucose, or both) comparing lifestyle curriculum plus stepwise addition of metformin (500 mg, twice daily) for participants at highest risk of conversion to diabetes to standard of care. After 3 years of follow-up, stepwise diabetes prevention in people with prediabetes can reduce diabetes incidence by a third in community settings (relative risk reduction = 32%, 95% CI = 7–50%) (46).

Reviewed prevention models are summarized in Table 1.

**Table 1:** Reviewed diabetes prevention models.

**Model**	**Brief description of model**	**Region**	**References**
The Da Qing IGT and Diabetes Study	Diet, exercise or combination 6 years maximum follow up	China	(38)
The Indian Diabetes Prevention Programme	Individually tailored lifestyle modification by mobile phone messaging 24 months follow up	India	(39, 40)
Lifestyle Modification in Information Technology (LIMIT)	Lifestyle modification in information technology consisting of mobile phone and e-mail (virtual assistance-based lifestyle advice) 1 year follow up	India	(41)
Kerala Diabetes Prevention Program	Community-based peer-support program of lifestyle change comprising 11 peer led group sessions, 2 diabetes prevention education sessions and participation in community activities 24 months maximum follow up	India, Kerala state	(42)
Nutrition Education Program (NEP)	Individual and group nutritional counseling from a team of nutritionists. 12 months follow up	Brazil	(43)
Community-based physical activity and nutrition program	Nutrition and physical activity program 6 months maximum follow-up	Vietnam	(44)
Diabetes Community Lifestyle Improvement Program (D-CLIP)	A step-wise model of diabetes prevention with lifestyle and metformin added when needed 3 years follow up	India	(45, 46)

In 2019, a systematic review and meta-analysis was published reporting on the effect of community-based programs on diabetes prevention in LMICs (47). It included all RCTs published in the last 10 years (from January 2008 to March 2018), including individuals with no diabetes ≥18 years of age at risk of T2DM who lived in LMICs and evaluating community-based programs/interventions for the prevention or risk reduction of T2DM (with no pharmacological intervention) compared with no program or standard health care advice. It was concluded that community-based interventions can modify several risk factors for T2DM, both anthropometric indices [weight, body mass index (BMI), and waist circumference] and glycemic control indices (fasting blood glucose and HbA_1c_). Moreover, the findings indicated that intervention resulted in lowering the risk of developing T2DM with a relative risk reduction of 0.57 (95% CI = 0.03–1.06). The small number of studies included and the heterogeneity of methods used resulted in wide confidence intervals, limiting the generalizability of results. As the participants' ages ranged from 30 to 76 years, the applicability of results to younger adults is also limited. Although T2DM is relatively rare among younger ages, recent reports indicate an increasing prevalence of T2DM in children and adolescents around the world. Currently, in the United States, the overall prevalence of T2DM among American youths aged 10 to 19 years rose by 35% (48) with a disproportionate representation in ethnic minorities. This trend is not limited to the United States, but is occurring internationally as well (49).

Consequently, more RCTs with longer follow-up duration in many LMICs, including younger ages, are needed to confirm the results and identify the most effective community-based model of intervention for prevention on risk reduction of T2DM.

Moreover, because the commonly used screening tools such as HbA_1c_ or oral glucose tolerance tests may be unavailable in low-resource settings, there is a need for cost-effective tools to identify individuals at risk of developing diabetes and its complications. Risk assessment tools such as FINDRISC in Colombia (50) or the Indian Diabetes Risk Score (51) in India have been used to identify individuals at high risk of developing diabetes and have been shown to be cost-effective in diabetes prevention.

## Models of Care

Patients with diabetes of LMICs, apart from the increased risk of late diagnosis, are at increased risk of not receiving optimal care leading to poor diabetes control and the development of diabetes-related complications (52). In many LMICs, there is lack of personnel and properly equipped clinics. Consequently, patients with diabetes often experience difficulty in accessing and affording standard and subspecialty health care. Hence, patients with diabetes in LMICs achieve the recommended targets for glycemia, blood pressure, and cholesterol in <10% of the time (10), with a higher proportion of premature deaths due to high blood glucose occurring in LMICs than in HICs (7). There is a clear need for effective models to deliver diabetes care designed for LMICs that account for the multitude of barriers to care in these low-resource health care settings.

At first, efforts were targeted to ameliorate the already existing models and structures. In order to support the care provided in primary care clinics, various educational and training programs have been initiated. In this context, the Step-by-Step Program in India and Tanzania aimed at training care providers in the management of diabetic foot. The program was established and instituted through collaboration between international organizations and academic centers. One physician and one nurse from each of 100 sponsored health care centers across India and Tanzania were assigned to attend lectures, practical demonstrations, problem-solving exercises, and hands-on experiences and were trained in the use of an algorithm for management of diabetic feet developed specifically for India and Tanzania (11, 12). More than 15,500 patients were screened across Tanzania, and the implementation of this program led to a significant reduction in foot complications from 24 to 8% and in amputations from 22 to 10%. The establishment of permanent foot clinics and the practical hands-on training of health care providers were obvious strengths of this program. To date, the model has been exported to various other countries in Africa (Democratic Republic of Cong, Guinea, Botswana, Malawi, Kenya, Ethiopia, Egypt, and Zimbabwe), Pakistan, Saudi Arabia, and the Caribbean (Barbados, St. Lucia, St. Maarten, St. Kitts, and the British Virgin Islands) (13). Similarly intensified nationwide training programs for health care providers and patients were applied in India. Specifically, the Rural and Semi-urban Diabetes Prevention and Control Program, the Indian Diabetes Educator Project, and the Distance Education in Diabetes Mellitus Program are nationwide training programs developed from the Department of Endocrinology, Diabetes, and Metabolism at Christian Medical College (CMC) in Vellore, India, in collaboration with academic and research centers and international organizations. To date, the program has been successful in terms of personnel trained and diabetes clinics established. Approximately 100 hospitals have already received training, 89% of which have diabetes clinics, and 95% of them use glucometers on a regular basis compared to 20% prior to initiation of the program, whereas 74% of them were enrolled in CMC Vellore's quality control program for monitoring the accuracy of basic biochemical tests related to diabetes (14, 15). In Ghana (16) and South Africa (24, 25), training programs aimed mainly at educating specialized primary care nurses to diagnose new patients and to refer them to specialists, as well as to apply initial treatment regimen, according to a developed treatment algorithm. Community involvement with distal learning potential was among the strengths of this model. In Ghana, the implementation of the Ghana Model for 3 years (16) led to trained diabetes health care teams in all regional and ~63% of subregional/district health facilities and established corresponding diabetes clinics/services for 10 tertiary/regional health facilities and 63 district hospitals in the country. Diabetes care guidelines were standardized. In South Africa, the Chronic Disease Outreach Program commenced in 1999 in Soweto (acronym for South Western Township, located southwest of Johannesburg) and was designed to follow patients with DM and hypertension, support primary health care nurses, and improve health systems for management. Nurses were directed to treat uncontrolled diabetes with insulin, proteinuria with angiotensin-converting enzyme inhibitors, and to add other medications as needed and directly refer patients for specialist care. A group of 257 DM patients and 186 nurses was followed over 2 years. Nurses successfully identified and treated 100% of patients and referred 95% of patients who needed further management. However, the sample was small, the follow-up poor, and the program could not integrate with the existing chronic disease service (24, 25). In order to ensure continuity, elements of the program have been integrated into the government- funded health care system. Staff turnover remains a significant difficulty in all these programs based on nurses' education and empowerment (26). Moreover, data on the effectiveness of these programs on diabetes care are not still available.

### Peers for Progress Programs

In several countries (Cameroon, Uganda, South Africa, and Thailand), programs focusing on peer support instead of health care providers were applied (27). In these programs, non-professional peer supporters received training in diabetes issues, in order to be able to assist in the management of a group of patients. Significant improvements in HbA_1c_ measurements have been reported, although sample sizes were small.

In Cameroon, 10 patients became peer supporters after training, and each peer supporter was assigned a group of 10 patients with whom he met regularly. After 6 months, mean HbA_1c_ decreased from 9.6 to 6.7%. In South Africa, 22 women participated, and all provided and received support from each other. In addition to paired session, discussions, and activities, pairs of participants used mobile phones to call and text each other messages with nutrition guidance and inducement to walk or get other exercise. In Thailand, 40 village health volunteers were trained in 2010 as peer supporters. Each worked with health centers to recruit three patients with diabetes. Data are available from 53 adults with T2DM who exhibited a significant decline in average HbA_1c_, from 8.6 to 7.9% (*p* = 0.027).

In Uganda, 46 participants and peer supporters were trained in diabetes self- management, and peer supporters received additional training in communication.

The participants' average diastolic blood pressure dropped from 85.39 to 76.27 mmHg, and average HbA_1c_ declined from 11.1 to 8.3%.

The evidence from the above projects was that peer support models can improve patients' metabolic control and quality of life, by promoting patients' daily health behaviors that are central to the management of diabetes.

### Automated Support Tools

An automated monitoring and self-care support tool was also tested in Hondura, Mexico, and Bolivia (17). Patients with diabetes reported health and self-care problems through their cell phones. Family caregivers and health care providers received updates about the patient status and information for supporting patient's diabetes management. The majority of patients (92%) were satisfied with the automated mobile health support and reported improved diet, medication compliance, and better perceived health. Mean HbA_1c_ levels improved by 1%. Moreover, the model tested a cloud-computing approach where calls were sent automatically to patients' mobile phones using interactive voice response (IVR). A total of 268 patients with diabetes or hypertension participated in 6 to 12 weeks of weekly IVR follow-up. Patient satisfaction was high, with 98% of patients reporting that the system was easy to use and 86% reporting that the calls helped them a great deal in managing their health problems (18). The same program was evaluated for the management of hypertension among patients with and without diabetes (19). Consequently, the University of Bolivia has worked in collaboration with University of Michigan to transfer this technology to a local platform, but results from this intervention program are still not available. This model demonstrated the utility of technology to facilitate care and the potential of its implementation in various Latin American countries.

In India, Shankhdhar et al. (28) tested the use of educational videos on a mobile phone, so-called mobifilms, which can be shared with patients and doctors via text message. One of the mobifilms has been put on the website of the International Diabetes Federation.

### Multicomponent and Integrated Care

In recent times, policy makers and health care providers are directed toward comprehensive care programs for patients with multimorbidity. These integrated programs are thought to increase system and cost-effectiveness, particularly in low-resource settings (53). Hence, programs integrating several diabetes care components have been evaluated in LMICs.

In the CARRS Trial in South Asia (20), 1,146 T2DM patients were randomly assigned to receive usual care by doctors or multicomponent intervention involving electronic health record management with decision support software physician directed, but coordinated by non-physician health workers. A significantly higher number of the intervention participants achieved the primary multiple risk factor control (HbA_1c_ <7% and either blood pressure <130/80 mmHg or low-density lipoprotein <100 mg/dL) compared to those receiving usual care.

The Chunampet Rural Diabetes Prevention Project was implemented in India to provide integrated diabetes screening, prevention, and care through the use of telemedicine and personalized care (21). Telemedicine is particularly significant to India because of its vast geographical area and predominant rural population with consequent difficulty in availability and accessibility of medical care. A telemedicine van was used to screen for diabetes and its complications equipped with retinal photography, Doppler imaging, biothesiometry, and electrocardiography using a satellite to communicate with a rural diabetes center. Of the total 27,014 adult population living in 42 villages, 86.5% were screened for diabetes. A total of 1,001 patients with diabetes were screened for complications. The mean HbA_1c_ levels of patients with diabetes in the whole community decreased by 0.7% within 1 year. The project empowered rural people. Only <5% of patients needed referral to the diabetes center, suggesting that 95% of the health problems of diabetes can be managed locally with considerable savings from the costs of transportation and treatment in the city.

Following the success of the DOTS (Directly Observed Therapy, Short course Model at treating tuberculosis, a similar short-course model was implemented in the diabetes clinic of Queen Elizabeth Central Hospital, the largest hospital in Blantyre, Malawi (22). The intervention consisted of care delivered from trained diabetes nurses using standardized diabetes treatment guidelines and increase of diabetic drugs supplies. In addition, an electronic medical record (EMR) was developed to create a registry and facilitate data analysis. Patients were equipped with a health passport with all important medical information and barcodes allowing rapid access to EMRs. The program was planned to expand to a national health monitoring system and include other chronic diseases. The analysis of EMRs with data recorded from more than 2,000 patients with diabetes over 3 years emphasized the potential for electronic records to facilitate clinical care, monitoring, and evaluation. However, it was also pointed out that physicians using electronic systems need ongoing training and that it is necessary to increase the number of touchscreen workstations to avoid missing data. Complications were not systematically screened, partly because of shortages of consumable materials, with fewer than 10% of subjects having any information recorded on complications. Regarding glycemic control, the trajectories of blood glucose levels from time since registration were examined within patient groups. The 25th, 50th (median), and 75th percentiles of blood glucose level within the patient group within 4-week groupings of time since registration were calculated, and for each percentile group (25th, 50th, and 75th), the trajectory of blood glucose values over time was plotted. Newly diagnosed cases seemed to make steady progression to glucose control, which was maintained thereafter, whereas previously diagnosed subjects had overall worse control, taking longer to get to glucose <150 mg/dL with some deterioration in control later. Patients aged ≥45 years required longer time to gain adequate control, but thereafter used to maintain more consistent glycemic levels than those aged <45 years. Subjects of normal or low BMI (<25 kg/m^2^) presented with higher blood glucose and needed more time to attain control compared to the overweight (BMI >25 kg/m^2^) who presented with lower initial glucose values, achieved earlier glucose control, and maintained it over time. It was assumed that different patient groups, particularly younger patients aged <45 years with low BMI <25 kg/m^2^, may require a different approach with more intensive support to medication adherence, whereas long-term diabetics would benefit more from an escalation in treatment as control deteriorates. The need for modified protocols for different patient groups was recognized (23).

In Shanghai, China, in an RCT, an integrated intervention program consisting of intensified education with frequent clinic visits on 150 patients with diabetes was compared to basic diabetes education. After 24 weeks, the intervention group had significantly lower HbA_1c_ (*p* < 0.001) (29). Similarly, In Costa Rica, an RCT on 75 patients with diabetes compared the results of a community-based nutrition and exercise program vs. basic diabetes education (30). A significant improvement in HbA_1c_ (*p* = 0.028) in the intervention group compared to the control group was reported. The implementation of this program demonstrated that even short-term but intensified education interventions can lead to significant control improvements. Moreover, Costa Rica has produced a distinct approach by implementing universal health coverage with unified financing, placing primary health care at the center of its health services network and focusing on care for leading cardiovascular risk factors including diabetes (54). The Costa Rican STEPS program established and adopted national guidelines for care of patients with diabetes, hypertension, and dyslipidemia, a national screening program with regular risk factor surveillance and provision of medications to health care centers. At the level of the health system, factors such as universal health coverage and availability of diagnostic tests and medications are important targets for countries looking to improve their health system for diabetes. Reviewed models-of-care programs are summarized in Table 2.

**Table 2:** Reviewed diabetes care models.

**Model**	**Brief description of model**	**Region**	**References**
Step by Step Diabetic Foot Project	• Collaboration between the International Diabetes Federation, International Working Group on diabetic foot, Diabetic Foot Society of India and Muhimbili University of Health and Allied Science. • Training of care providers in the management of diabetic foot based on established algorithm of diabetic foot care	India and Tanzania	(11, 12)
Rural and Semi-Urban Diabetes Prevention and Control Program Indian Diabetes Educator Project Distance Education in Diabetes Mellitus Program	• Collaboration between Department of Endocrinology, Diabetes, and Metabolism at Christian Medical College (CMC) in Vellore, India with World Diabetes Foundation, Project HOPE, the Christian Medical Association of India, the Schieffelin Leprosy Research Training Center, and Albert Einstein College of Medicine. • Training of personnel and establishment of diabetes clinics • Community education and screening programs	Vellore, India	(15)
The Ghana model	• Collaboration between academic centers and local government • Training of diabetes health care teams and establishment of diabetes clinics • Standardization of diabetes care guidelines	Ghana	(16)
The Chronic Disease Outreach Program (CDOP)	• Collaboration between academic centre and local government • Training of nurses in diabetes treatment and referring patients appropriately	Soweto, South Africa	(24, 25)
Peers for Progress Programs	• Training non- professional peer supporters in diabetes issues • Peer supporters based in diabetes clinics (Cameroon and Uganda), community organizations outside the field of health (South Africa), and a volunteer service part of the health system (Thailand) • Use of technology	Cameroon, Uganda, South Africa, and Thailand	(27)
Automated support tools	• Collaboration between academic centers, telecommunication companies and health authorities • Use of technology	Honduras, Mexico, Bolivia	(17–19)
CARRS Trial	• Multi-component intervention involving electronic health record management with decision support software (EHR-DSS) • Coordination by non-physician health workers	in South Asia	(20)
Chunampet Rural Diabetes Prevention Project (CRDPP)	• Integrated diabetes screening, prevention and care through the use of telemedicine and personalized care	India	(21)
Directly Observed Therapy, Short course) Model (DOTS)	• Collaboration between academic centers and international organizations • Electronic medical record • Establishment of national diabetes register	Malawai	(22)
Integrated Intervention Program	• Intensified patient education	Shangai, Costa Rica	(29, 30)

## Health Care Models’ Performance

Most of the reported models lack evaluation of performance. There is a need for evidence on health care models' performance in order to disseminate their application, design targeted policies, and constitute a reference point in the future. By evaluating the system's performance preferably using the cascade of care analysis—a quantitative portrait of the stepwise care system involving testing, diagnosis, treatment, adherence to treatment, and effective control—it is possible to identify at which stage of the system the largest losses to care occur. Data on health systems' performance in LMICs are limited. In a national representative sample of population (4,083 total subjects, of whom 521 had diabetes) from South Africa, 45% of people with diabetes had not been screened, 15% had been screened but not diagnosed, and 18% of those who had been treated were not adequately controlled (55).

In a study from Seychelles, at national level in a random sample of 1,255 participants, it was reported that only 54% of people with diabetes were aware of the diagnosis, and while treatment was offered in 98% of them, only 21% had satisfactory diabetes control (56). A population-based study in Malawi, enrolling 28,891 adults (≥18 years) residing in two defined geographical areas, also reported 59% of people with diabetes being diagnosed, out of whom 62% being treated and only 41% of those under treatment achieving adequate control (57).

In 2019, an analysis of health systems' performance for management of diabetes across 28 LMICs in multiple geographic regions from nationally representative population-based surveys was performed. In this analysis, data collected from 2008 onward were included and analyzed by cascade of care analysis. The authors overall confirmed the previous results (58). They also reported that health system performance for diabetes management in low-resource settings is generally characterized by large losses to care at the stage of diabetes testing and only moderate rates of diabetes control. The large losses to care at the stage of diabetes testing can be partly attributed to the lack of clear global guidelines for screening and screening activities in LMICs and represent a challenge for the health systems. A systematic review to compare type 2 DM guidelines in individual LMICs vs. HICs reported that most of LMIC guidelines were inadequate in terms of clinical applicability, clarity, and rigorous dissemination plan, as well as socioeconomic and ethical–legal contextualization. The need for broad-based guidelines recommending clear up-to-date clinical interventions carefully contextualized with respect to specific sociocultural and economic barriers and facilitators was emphasized (59).

On the other hand, the losses relating to achieving glycemic control are less explained by inadequacy of health systems' resources and mostly linked to barriers to patients' adherence. There are several factors that may affect the level of adherence Apart from the limited access to support services, which has been reported as a leading health system's barrier to adherence and lack of health insurance, medicines, and affordability of dietary recommendations (60), there are difficulties of changing long-term behaviors or changing food choices and eating habits based on cultural meaning of food, social pressure, and so on (61, 62). This finding underlines the need for understanding the barriers and facilitators of patients' attitudes toward self-care in order to design population-adjusted approaches to influence human behavior.

Another interesting finding from this analysis was that younger or underweight individuals with diabetes in these settings had worse performance than older and overweight/obese individuals in terms of rates of testing, service utilization, and diabetes control. This finding indicates the need for especially guiding screening and reinforcing linkage to care in these particular subsets of populations.

Moreover, among patients with diabetes diagnosed and treated, only a percentage of 18% of those receiving lifestyle modification advice as therapy achieved control, whereas 58% of those treated with medications with no lifestyle modification advice achieved control. This finding indicates that in these populations lifestyle advice alone, most of the time, may not be sufficient for achieving control.

Further follow-up of these indicators in combination with cost-effectiveness analyses (63) and multicriteria decision analysis (64) is needed in order to plan targeted interventions and policies and allocate resources within health systems. The economic evaluation of health care policy in LMICs is of great importance, considering the resource limitations in these countries. Guidelines and data resources for such economic evaluation in LMICs are available (65). Adherence to these guidelines and standardization of research methods are needed for a high-quality economic evaluation, which is important for effective decisions from policy makers and health care organizations.

In conclusion, during the last years, we are observing an epidemiological transition in LMICs. Non-communicable diseases such as DM are predicted to overtake infectious diseases, maternal/infant mortality, and malnutrition by 2030 (66). There is a critical window for the development and implementation of effective diabetes care models in LMICs. These models can serve as a stimulus for the change in delivery and receiving medical care in LMICs, and diabetes can serve as a potential tracer for reexamining health systems in these countries. Diabetes health care models should target increasing patients' access to care, training patients and local health care professionals, standardizing guidelines for prevention and management, and using technological innovation to improve the effectiveness of care provided and health outcomes globally.

## Author's Note

There is a growing epidemic of diabetes in the world and two thirds of diabetes patients live in middle and low income countries. The prevalence of diabetes is increasing especially among people with poor economic status and malnutrition. There is a critical window for the development and implementation of effective diabetes care models in low income settings. Data on this topic are limited in the literature. In this paper we aim to describe the latest evidence on healthcare models in LMICs for preventing/delaying the onset of the disease in high risk individuals and for controlling the disease when already present. We also try to identify the features and qualities that can make these programs successfully implemented, scaled up and sustained in the diabetes care in these low resource contexts.

## Author Contributions

GS was responsible for the acquisition and analysis of the data. AS comtibuted in drafting and writing. FK was responsible for the design of the work and contributed in writing. All authors listed have made a substantial, direct and intellectual contribution to the work, and approved it for publication.

## Conflict of Interest

The authors declare that the research was conducted in the absence of any commercial or financial relationships that could be construed as a potential conflict of interest.
